# Genome-wide identification and comparative analysis of Dmrt genes in echinoderms

**DOI:** 10.1038/s41598-023-34819-z

**Published:** 2023-05-11

**Authors:** Quanchao Wang, Tiangui Cao, Yanxia Wang, Xiaojing Li, Yue Wang

**Affiliations:** 1 Key Laboratory of Coastal Biology and Bioresource Utilization, Yantai Institute of Coastal Zone Research, Chinese Academy of Sciences, Yantai, 264003 China; 2 Key Laboratory of Ecological Warning, Protection and Restoration for Bohai Sea, Ministry of Natural Resources, Qingdao, 266061 China; 3 Yantai Vocational College, Yantai, 264670 China

**Keywords:** Evolution, Genetics

## Abstract

The Dmrt (Doublesex-mab3-related transcription factor) gene family is a class of crucial transcription factors characterized by one or several conserved DM (Doublesex/Mab-3) domains. Dmrt family genes can participate in various physiological developmental processes, especially in sex determination/differentiation. Echinoderms are extremely important research objects in various fields, such as sex determination/differentiation and neuroscience. However, to date, the genome-wide characterization and analysis of Dmrt genes in echinoderms have not been investigated. In this study, the identification and analysis of Dmrt genes in 11 representative echinoderms were performed using bioinformatics methods. A total of 43 Dmrt genes have been found in the studied echinoderms, and the number of Dmrt genes in different species ranges from 2 to 5. The phylogenetic tree showed that all Dmrt genes from echinoderms can be subdivided into 5 classes, the Dmrt2-like class, Dmrt3-like class, Dmrt4/5-like class, Dsx-like class, and a novel Dmrt (starfish-specific) class. Furthermore, selective pressure assessment suggested that the Dmrt genes underwent purifying selection pressure. In general, this study provides a molecular basis for echinoderm Dmrt genes and may serve as a reference for in-depth phylogenomics.

## Introduction

The Dmrt (Doublesex-mab3-related transcription factor) gene, including one or several DM (Doublesex/Mab-3) domains, has been widely studied due to various functions^1–4^, especially in sex determination/differentiation. For example, the Z-linked Dmrt1 gene is vital for sex determination/differentiation in birds^5^. In *Xenopus laevis*, a W-linked Dmrt gene can be involved in the development of the primary ovary^6^. A Y-specific Dmrt gene, DMY/Dmrt1bY, can determine the sex of *Oryzias latipes*^7–9^. In general, the Dmrt gene family is an important gene family involved in sex-related development during organism evolution.

To date, Dmrt family genes have been studied in mammals, teleosts, and insects. The members of the Dmrt gene family showed substantial differences in different organisms. For example, eight Dmrt members have been found in some mammals^4^, such as *Homo sapiens* and *Mus musculus*. In teleosts, a total of seven Dmrt members have been identified from *Larimichthys crocea*^10^, and five Dmrt genes have been found in *Oreochromis niloticus*^11^. In *Drosophila melanogaster*, only four Dmrt genes have been identified. However, to date, no genome-wide study has been conducted to identify Dmrt genes in echinoderms.

Echinoderms have usually been considered to be the closest invertebrate sister group of vertebrates^12^, with particular evolutionary classification and phylogeny. Meanwhile, as an ancient invertebrate group, echinoderms have diverse reproduction modes, including asexual multiplication, parthenogenesis, hermaphroditism, and dioecy^13^. Therefore, echinoderms are extremely important research objects in many fields, such as sex determination/differentiation^13^, neuroscience^14^, and regeneration biology^15^. In particular, recent studies have suggested that some biological processes in echinoderms are associated with Dmrt genes^16,17^. However, no research has focused on the systematic investigation of Dmrt family genes in echinoderms.

The main research objective of this study was to systematically analyze the abundance of Dmrt genes in echinoderms. With the decoding of many echinoderm genomes, including *Acanthaster planci*^18^, *Anneissia japonica*^19^, *Apostichopus japonicus*^20^, *Asterias rubens* (NCBI: PRJNA683060), *Hemicentrotus pulcherrimus*^21^, *Holothuria glaberrima*^22^, *Lytechinus variegatus*^23^, *Patiria miniate* (NCBI: PRJNA683060), *Plazaster borealis*^24^, *Strongylocentrotus purpuratus*^25^, and *Temnopleurus reevesii*^26^, genome-wide identification and analysis of the Dmrt gene family was carried out. Furthermore, the functional domains and sequence structures of the Dmrt genes were predicted, and phylogenetic analysis was carried out. The findings from this study can provide fundamental insights into the evolutionary and physiological aspects of Dmrt genes in invertebrates.

## Materials and methods

### Sequence identification

A set of DMRT protein sequences within different species was first obtained from NCBI databases, including *H. sapiens*, *M. musculus*, *Macaca fascicularis*, *Balaenoptera musculus*, *Gallus gallus*, *O. latipes*, *O. niloticus*, *L. crocea*, *D. melanogaster*, *Bombyx mori*, *Aedes aegypti, Sagmariasus verreauxi and Cherax quadricarinatus* (Supplementary Table S1). The Dmrt genes in 11 echinoderms were identified by combining BLAST and HMM search strategies. First, the genome and annotation files of 11 echinoderms were downloaded from different genome databases (Supplementary Table S2), and the DM domain query (accession: PF00751) was downloaded from Pfam (http://pfam.xfam.org/). Second, BLAST V2.11.0^27^ and HMMER V3.2.1^28^ were used simultaneously to search DMRT proteins in all genomes with the DM domain query. The initial E-values for both the BLAST and HMM searches were set to 1 × 10^–5^ and 1.0, respectively. Third, the candidate genes obtained by BLAST and HMM searches were merged, and then the redundant genes were removed. Finally, to confirm the existence of the DM domain, the nonredundant genes were further checked by using the NCBI CDD database according to an E-value of 10^–5^. When multiple transcripts were annotated for a gene, the longest transcript was selected. The properties of Dmrt proteins in echinoderms were predicted by using TBtools v1.098^29^.

### Phylogenetic analyses of the Dmrt gene family

All retrieved Dmrt proteins from NCBI and those identified from 11 echinoderms were utilized to perform the phylogenetic analysis. Multiple sequence alignments of Dmrt proteins were first generated using MAFFT v7.310^30^. Then, the phylogenetic trees were constructed by IQTREE v2.1.2^31^ with the following settings: -m MFP -bnni -B 2000 -T AUTO. The phylogenetic trees were displayed using iTOL (interactive tree of life) online tool^32^.

### Sequence analyses and genomic distribution of Dmrts

The general feature format file was used to reveal the Dmrt gene structure and exon information. The conserved motifs of the Dmrt genes were predicted by using MEME^33^ with the following options: largest number, 25; minimum length, 6; maximum length, 50; and default values for other parameters. Conserved motifs and gene structure were both visualized by TBtools v1.098^29^. In addition, the conserved domains of all identified DMRT genes were analyzed using the Batch SMART plug-in in TBtools software (version 1.098)^29^ and visualized with the iTOL (interactive tree of life) online tool^32^. The genomic distribution was visualized with gene arrow maps generated by using the gggenes package in R^34^.

### Selective pressure assessment

Selective pressure was assessed by using the branch and site model in EasyCodeML V1.0^35^. The branch models assume that the ratios (ω) of nonsynonymous substitution sites (dN) and synonymous substitution sites (dS) vary among branches. Under the branch model, the comparison of two models (one ratio and free ratio) was calculated to test whether ω is different among different branches. The site models assume that the ω ratio varies among sites. Under the site models, the specific models (M0, M1a, M2a, M3, M7, and M8) were tested by adjusting the parameters. Among these models, comparison of M3/M0 is used to detect whether the ω ratio between different sites is consistent, while the comparisons of the M2a/M1a and M8/M7 model pairs test for positive selection.

## Results

### Identification and characterization of Dmrt genes

A total of 43 Dmrt genes have been identified in 11 representative echinoderms. The amino acid sequences of the identified Dmrt genes are provided in Supplementary Table S3. The number of Dmrt genes in each species ranges from 2 to 5, which are listed in Table 1. The characteristics of all the identified proteins in echinoderms were predicted and are listed in Table 1. The results showed that the biophysical properties of different Dmrt proteins were different. AA length varied from 110 to 794. The MW ranged from 12,660.77 to 88,920.97 Da, while the PI values varied from 5.44 to 10.37. Additionally, the vast majority of Dmrt proteins were considered unstable (instability index greater than 40).

**Table 1:** Protein sequence features of identified Dmrts in echinoderms.

Species	Gene ID	Protein ID	AA	MW	PI	INS	AIN	GRAVY
*Hemicentrotus pulcherrimus*	Hp-HPU_09801	HPU_09801	490	52,774.33	5.91	50.48	65.8	− 0.591
	Hp-HPU_09660	HPU_09660	535	58,708.26	9.42	52.91	66.69	− 0.673
	Hp-HPU_07004	HPU_07004	731	82,013.79	6.03	65.3	56.17	− 0.747
*Strongylocentrotus purpuratus*	Sp-XP_786938.3	XP_786938.3	503	53,511.34	9.2	64.66	60.62	− 0.548
	Sp-XP_030852253.1	XP_030852253.1	469	50,653.07	6.16	52.18	66.44	− 0.576
	Sp-XP_030851643.1	XP_030851643.1	469	51,257.85	9.83	50.5	62.58	− 0.751
	Sp-XP_030854341.1	XP_030854341.1	708	79,043.58	5.44	72.07	55.51	− 0.63
	Sp-XP_030839690.1	XP_030839690.1	708	78,950.5	5.56	70.53	56.06	− 0.616
*Lytechinus variegatus*	Lv-XP_041474728.1	XP_041474728.1	230	25,739.31	9.51	59.68	64.91	− 0.648
	Lv-XP_041474858.1	XP_041474858.1	522	57,326.55	9.72	45.77	64.48	− 0.779
	Lv-XP_041475654.1	XP_041475654.1	471	50,940.25	6.23	50.98	65.12	− 0.615
	Lv-XP_041467722.1	XP_041467722.1	784	85,754.54	5.76	63.8	62.36	− 0.549
*Temnopleurus reevesii*	Tr-TRE_26769	TRE_26769	110	12,660.77	10.24	28.94	80	− 0.697
	Tr-TRE_05017	TRE_05017	465	51,195.95	9.69	61.2	70.54	− 0.696
	Tr-TRE_16693	TRE_16693	794	88,920.97	5.84	77.83	59.7	− 0.723
*Apostichopus japonicus*	Apj-PIK34621.1	PIK34621.1	216	23,931.91	8.83	49.88	59.68	− 0.896
	Apj-PIK44057.1	PIK44057.1	314	34,485.65	7.06	54.64	61.59	− 0.753
	Apj-PIK33706.1	PIK33706.1	168	18,852.66	9.81	50.61	65	− 0.511
	Apj-PIK41536.1	PIK41536.1	282	32,902.18	8.72	76.93	47.66	− 1.033
	Apj-PIK43860.1	PIK43860.1	342	38,810.81	9.41	77.68	73.57	− 0.79
*Holothuria glaberrima*	Hg-Hglab.06749g1.t1	Hglab.06749g1.t1	165	18,658.19	9.1	50.95	60.36	− 0.741
	Hg-Hglab.14937g1.t1	Hglab.14937g1.t1	144	16,029.33	8.84	41.65	66.46	− 0.665
	Hg-Hglab.00147g3.t1	Hglab.00147g3.t1	415	46,125.35	9.96	59.05	68.14	− 0.683
	Hg-Hglab.05111g1.t1	Hglab.05111g1.t1	270	30,902.68	8.24	56.72	43.33	− 1.025
*Acanthaster planci*	Ap-XP_022089262.1	XP_022089262.1	464	50,585.04	9.07	59.13	60.8	− 0.631
	Ap-XP_022087749.1	XP_022087749.1	469	51,102.62	9.96	52.97	65.78	− 0.629
	Ap-XP_022089263.1	XP_022089263.1	432	46,167.22	8.81	57.86	61.5	− 0.458
	Ap-XP_022111791.1	XP_022111791.1	481	50,994.01	9.3	60.11	58.25	− 0.608
*Asterias rubens*	Ar-XP_033636556.1	XP_033636556.1	463	50,628.15	9.22	60.05	60.32	− 0.619
	Ar-XP_033636851.1	XP_033636851.1	419	46,299.06	10.01	57.13	59.36	− 0.744
	Ar-XP_033637002.1	XP_033637002.1	428	45,748.68	8.74	55.51	61.19	− 0.458
	Ar-XP_033629679.1	XP_033629679.1	441	48,419.31	8.57	54.39	59.07	− 0.576
*Patiria miniata*	Pm-XP_038069302.1	XP_038069302.1	467	50,940.38	9.08	58.76	63.94	− 0.62
	Pm-XP_038069487.1	XP_038069487.1	467	50,940.38	9.08	58.76	63.94	− 0.62
	Pm-XP_038048228.1	XP_038048228.1	456	49,684.93	9.93	47.2	58.44	− 0.743
	Pm-XP_038069303.1	XP_038069303.1	432	46,072	8.86	50.51	62.43	− 0.447
	Pm-XP_038049237.1	XP_038049237.1	497	54,098.74	9.34	58.31	59.07	− 0.661
*Plazaster borealis*	Pb-KPB_00015970-RA	KPB_00015970	468	51,121.44	9.74	61.64	63.31	− 0.699
	Pb-KPB_00012322-RA	KPB_00012322	425	46,252.49	8.42	56.98	56.28	− 0.626
*Anneissia japonica*	Anj-XP_033117096.1	XP_033117096.1	411	44,236.9	9.42	61.64	64.43	− 0.514
	Anj-XP_033124332.1	XP_033124332.1	360	40,734.98	10.37	47.08	72.06	− 0.614
	Anj-XP_033101076.1	XP_033101076.1	416	45,716.6	7.92	56.31	65.91	− 0.596
	Anj-XP_033105012.1	XP_033105012.1	238	26,432.9	9.18	67.26	52.48	− 0.665

*AA* amino acid length, *MW* molecular weight, KD, *PI* isoelectric point, *INS* instability index, *AIN* aliphatic index, *GRAVY* grand average of hydropathy.

### Phylogenetic analysis of Dmrt genes

**Figure 1:**
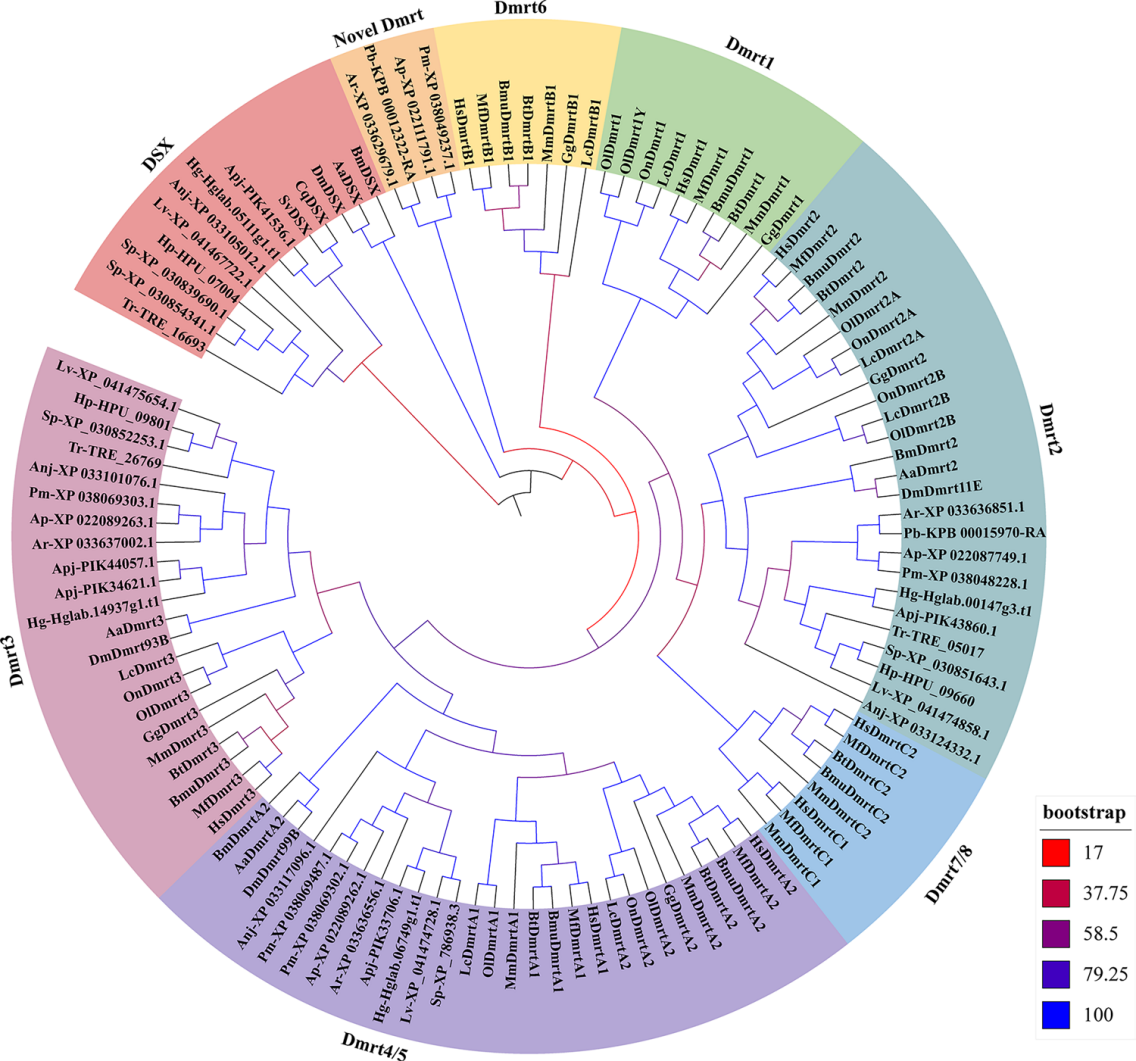
Phylogenetic tree of 118 Dmrt protein sequences.

To understand the evolutionary relationships of Dmrt genes in echinoderms, a phylogenetic analysis was carried out using Dmrt protein sequences from vertebrates and invertebrates. As shown in Fig. 1, 43 Dmrt genes from echinoderms were divided into 5 classes: the Dmrt2-like class, Dmrt3-like class, Dmrt4/5-like class, Dsx-like class, and novel Dmrt class. The Dmrt2-like class contains 11 genes from 11 echinoderms. The Dmrt3-like class consists of 11 Dmrt genes from 10 echinoderms. Nine Dmrt genes from 8 echinoderms have formed a Dmrt4/5-like class. The Dmrt genes in the Dsx-like class come from sea urchins, sea cucumbers, and crinoids. The remaining genes from starfish were divided into a novel Dmrt class.

### Sequence analyses and genomic distribution

**Figure 2:**
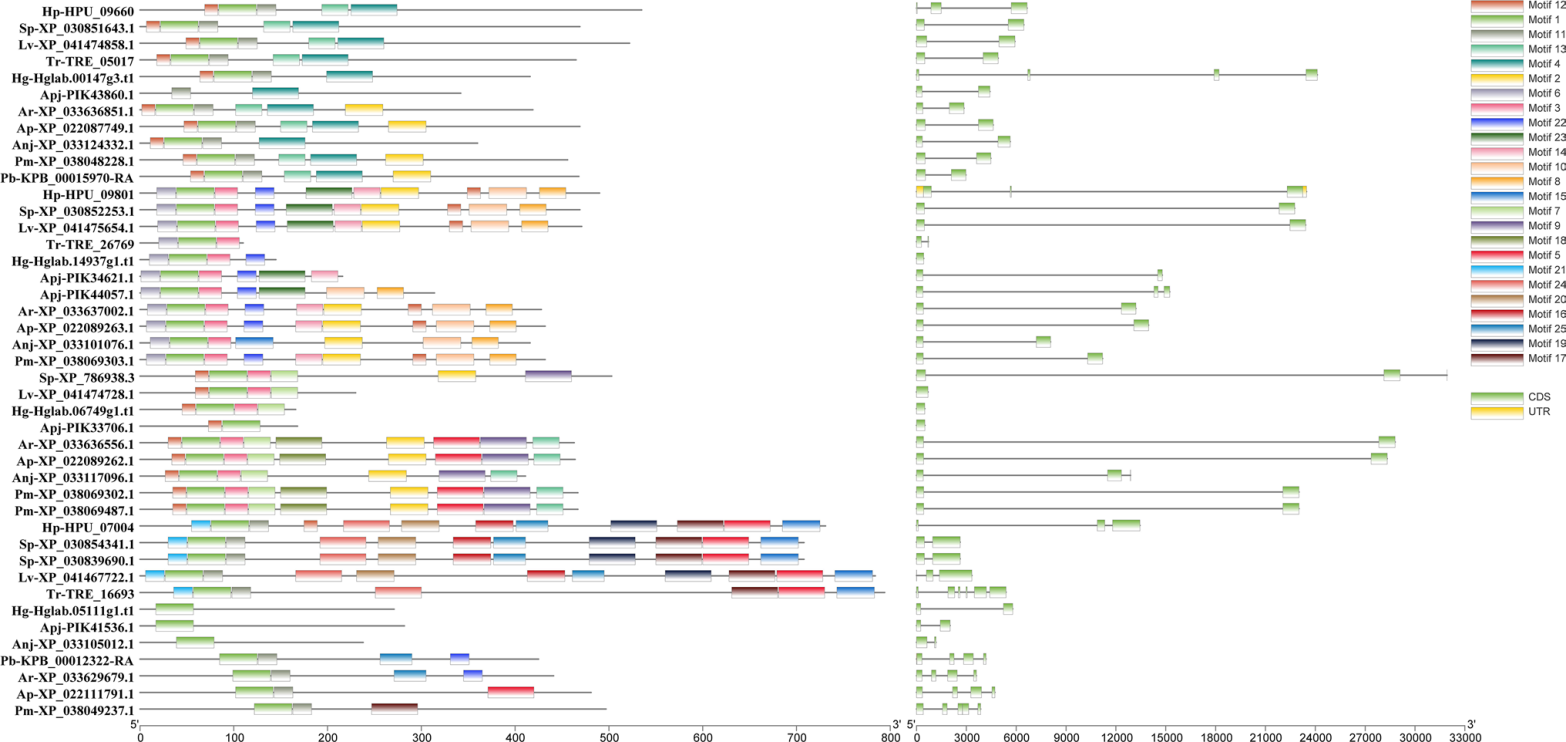
Motif composition and exon‒intron structures of echinoderm Dmrt genes.

**Figure 3:**
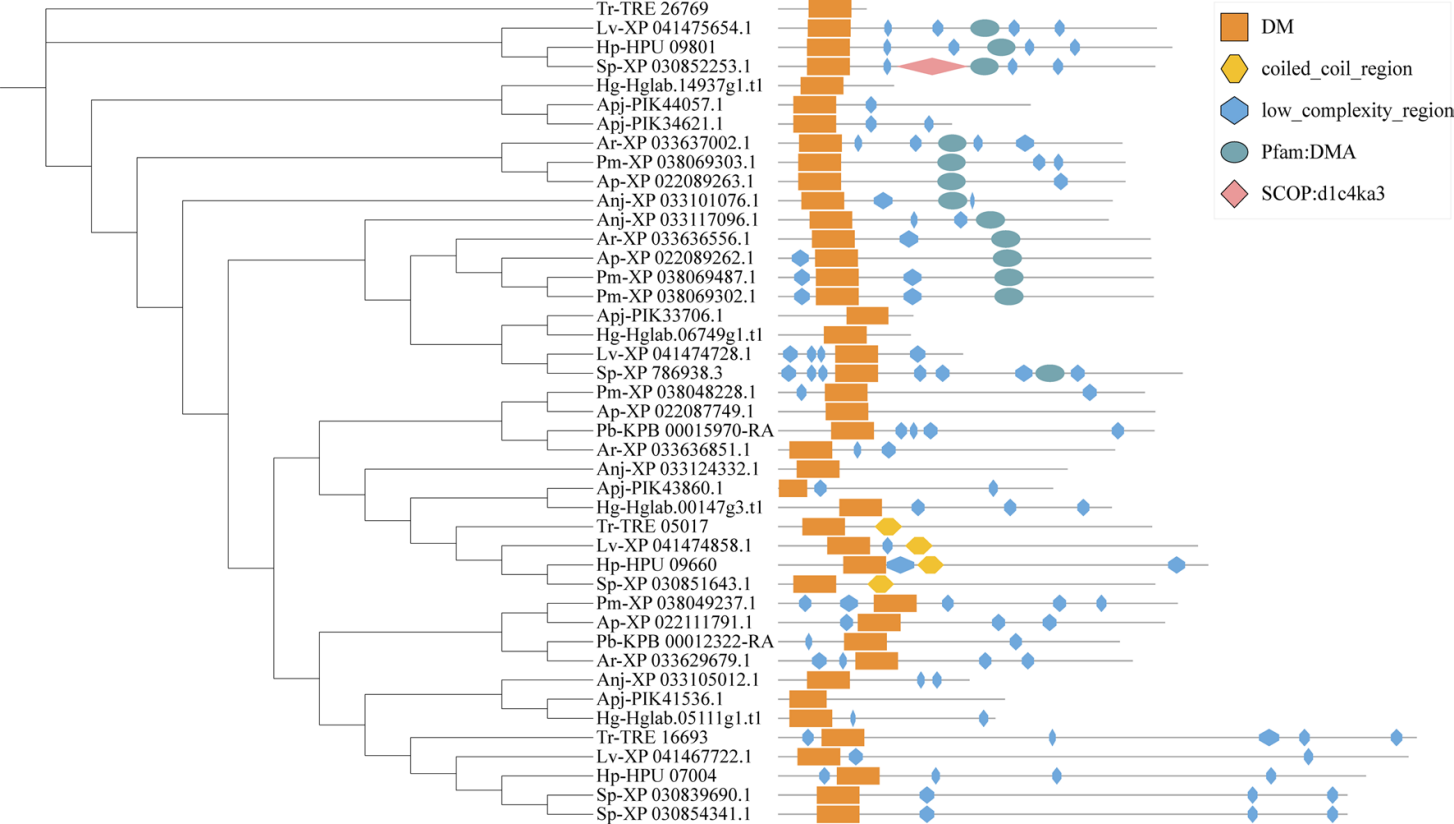
Conserved domain structures of Dmrt genes.

**Figure 4:**
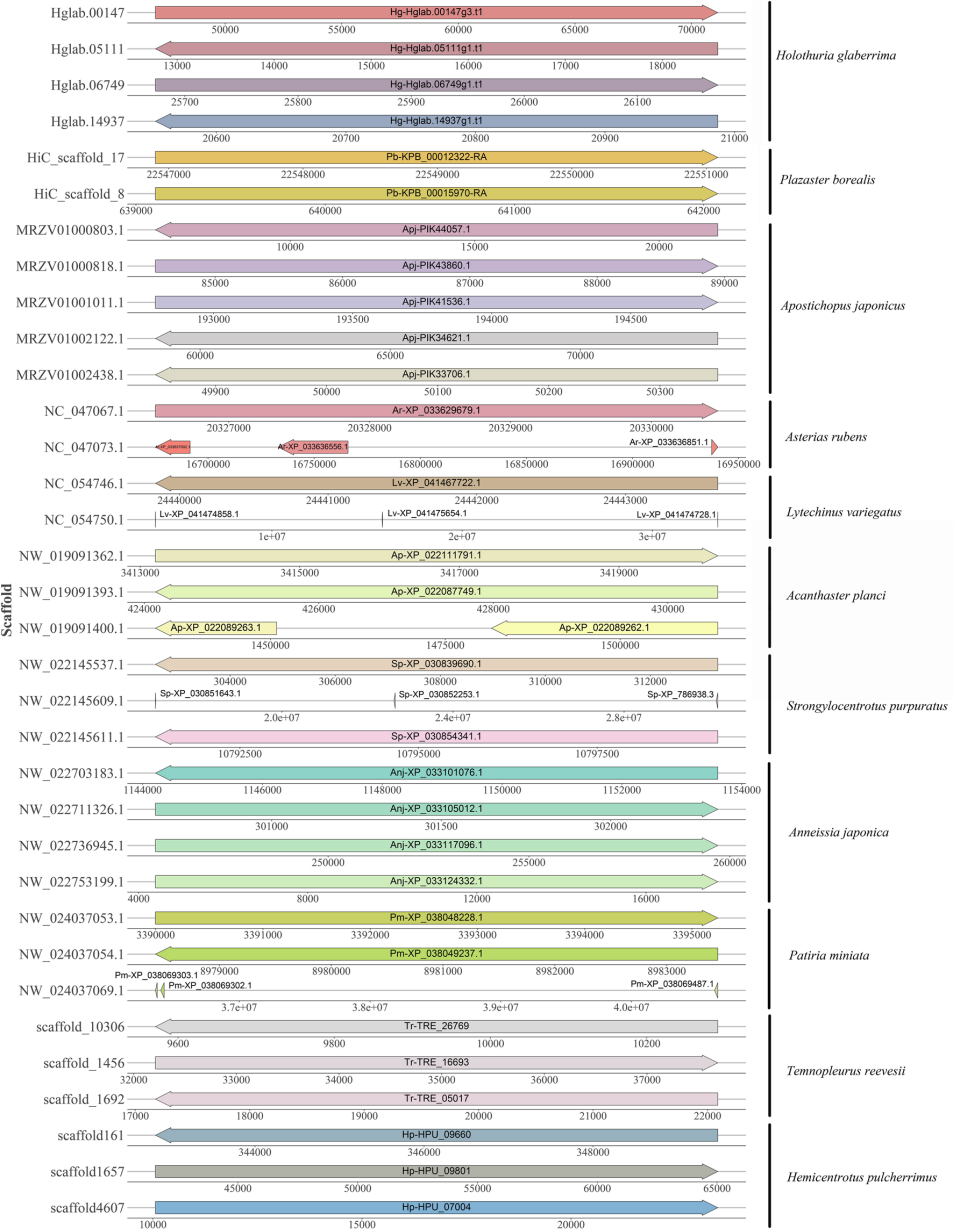
Genomic distribution of echinoderm Dmrt genes.

The exon‒intron diversification among echinoderm Dmrt genes is also displayed in Fig. 2. The exon numbers of Dmrt genes in 11 echinoderms varied from 1 to 6. Genes in the same class have more similar exon‒intron structures. In addition, although all the predicted Dmrt proteins contain motif 1, the proteins in the same class have more similar motif structural features. By using the Batch SMART search, it was found that all Dmrt genes include a DM domain, and some Dmrt genes contain Pfam:DMA (Fig. 3). The genomic locations of Dmrt genes in different species are shown in Fig. 4. The Dmrt gene cluster can be found in several species. The Dmrt2-like/Dmrt3-like/Dmrt4-like cluster can be found in *A. rubens*, *L. variegatus*, and *S. purpuratus*, while the Dmrt3-like/Dmrt4-like cluster can be found in *A. planci*. In addition, a Dmrt3-like/Dmrt4-like/Dmrt4-like cluster was identified in *P. miniata.* Dmrt genes in other echinoderms were randomly distributed on separate scaffolds, which may be due to incomplete genome assemblies.

### Selective pressure analysis

In the branch model, Model = 0 (M0) and 1 (M1) were chosen to test whether the ω values across different branches were significantly different (Table 2). The ω for the M0 model was 0.016. Comparison of the M1 model with the M0 model revealed that each branch has similar ω values (*P*_*LRT*_ = 0.976). Subsequently, the site model was used to identify the positive selection sites of the Dmrt gene of echinoderms. In the site model, the M3 model significantly outperformed the M0 model, suggesting that variable alternative pressure existed among different sites. However, no significantly positively selected sites were found in Dmrt by comparisons (M2a/M1a and M8/M7). Considering differences in terms of protein sequence, these results suggest that the homologous (or conserved) domain region of Dmrt genes underwent purification selection.

**Table 2:** Selection analysis of the Dmrt genes in echinoderms.

Model	Model type	np	LnL	ω	Model comparison	*P*_LRT_
Branch model	0	85	− 3376.007	0.016	M1/M0	0.976
	1	86	− 3376.007			
Site model	M0	85	− 3380.110	0.013	M3/M0	0.000
	M3	89	− 3282.014			
	M1a	86	− 3380.111		M2a/M1a	0.971
	M2a	88	− 3380.081			
	M7	86	− 3289.575		M8/M7	0.999
	M8	88	− 3289.576			

## Discussion

The Dmrt gene family has been identified genome-wide in various animal groups, including mammals, insects, and teleosts^10,36,37^. However, little is known about aquatic invertebrates. In particular, a comprehensive survey of Dmrt genes has not been carried out in echinoderms, although some echinoderm genome sequences have been available for several years. In the current study, a systematic analysis of Dmrt family genes was performed in 11 echinoderm genomes. Two to five Dmrt genes have been identified in different echinoderms. According to previous studies, the difference in the number of Dmrt genes may be related to genome size and genomic duplication rounds^37^. Although Dmrt genes are widely represented across the animal kingdom, they present a certain degree of species specificity. For example, Dmrt1 is found only in vertebrates, and Dmrt 6–8 is only present in mammals. This pattern was confirmed in this study. No Dmrt1-like gene or Dmrt6/7/8-like gene was identified in echinoderms, while Dmrt2-like, Dmrt3-like, Dmrt4/5-like and possibly Dsx-like genes were found in this study. Similar Dmrt members can also be found in Panarthropoda^38^. These results imply that Dmrt2, Dmrt3, Dmrt4/5, and Dsx-like genes may be widely present in invertebrates.

Unlike some fish that harbored two paralogs of Dmrt2 (Dmrt2a and Dmrt2b), all the studied echinoderms carried one Dmrt2-like gene, suggesting that the Dmrt2-like gene may be conserved in echinoderms. Dmrt2 has very important roles in sex reversal, testicular development, and embryonic development. For example, in humans, Dmrt2 is associated with XY sex reversal and gonadal dysgenesis^39^. Dmrt11E was proven to be a crucial factor for gametic formation in domesticated silkworm^40^. Analogous functions of Dmrt2 have also been reported in several aquatic invertebrates. In *Penaeus monodon*, Dmrt11E was proposed to affect muscle development, testis development, spermatogenesis, and somites^41^. In *Chlamys nobilis*, Dmrt2 is expressed exclusively in gonads, implying that it may be involved in the maintenance of gonadal function or gonadal development^42^. In addition, in zebrafish, Dmrt2 was found to have a function in regulating the left–right patterning of the mesoderm^43^. In summary, given that Dmrt2 has diverse functions, Dmrt2-like genes in echinoderms should be further studied.

In this study, both Dmrt2-like genes and Dmrt3-like genes were found in all species except *P. borealis*. Furthermore, the Dmrt2-like/Dmrt3-like cluster can be found in numerous echinoderms. These results may support the previous conclusion that Dmrt3 may have emerged through a gene duplication event of Dmrt2 during deuterostome evolution^44^. In addition, functional investigations on Dmrt3 have only been performed in vertebrates, showing that this gene can play pivotal roles in configuring the spinal circuits controlling stride^45^. Consistent with previous findings, the current phylogenetic analysis showed that Dmrt4 and Dmrt5 were clustered into a major branch, suggesting that these two types of genes originated from the same ancestor of Dmrt. In this study, the Dmrt4/5-like gene was identified in 8 echinoderms with the exception of *H. pulcherrimus*, *T. reevesii,* and *P. borealis*. In particular, the Dmrt4/5-like gene was duplicated in *P. miniata*. To date, Dmrt4 and Dmrt5 have been found to be closely related to neurogenesis. For instance, in *Xenopus*, Dmrt4 and Dmrt5 are important regulators of olfactory placode neurogenesis^46^. During the development of the hippocampus in mice, Dmrt5 was shown to be involved in the regulation of the neuron–glia cell-fate switch^47^. A similar function was also observed in invertebrates. In *Drosophila*, Dmrt99B plays an essential role in initiating temporal patterning in medulla neuroblasts. Thus, it will be interesting to investigate whether Dmrt4/5 play similar functions in echinoderms.

Dsx was found to have a pivotal role in sexual dimorphism in genetic sex-determining animals, including insects and nematodes. In *Drosophila*, Dsx has male- and female-specific isoforms (DsxM and DsxF), which can regulate different target genes, resulting in sex-specific morphology^48^. In *B. mori*, two Dsx isoforms (BmDsxF and BmDsxM) can enhance male and female differentiation in gonads and external genitalia, respectively^49^. DapmaDsx1 (Dsx ortholog) in *Daphnia magna* was confirmed as a critical regulator of the male phenotype^50^. In this study, a possible Dsx-like gene class was found in the phylogenetic tree. However, whether these genes have similar functions is unclear. In addition, it should be noted that the Dsx gene class in the phylogenetic tree was backed by low bootstrap values. In particular, these sequences from sea urchins, sea cucumbers, crinoids, and two Dsx sequences from shrimp seem to be unrelated to the Dsx from *A. aegypti*, *B. mori*, and *D. melanogaste*r. This result may be caused by relatively few informative characters outside of the DM domains. In particular, it is worth noting that the members in the Dsx-like cluster present different protein characteristics. For example, the AA and MW of the Dsx-like gene from sea urchins were significantly higher than those of the Dsx-like gene from other species. These results may imply that the function of Dsx-like genes in sea urchins may be different from that of Dsx-like genes in other species.

Moreover, a starfish-specific Dmrt class was identified in the current study. These genes were phylogenetically distant from the other Dmrt members. Their exon‒intron structure is also unique. Similar results can be learned in other aquatic invertebrates. Comparative phylotranscriptomics revealed that DMRT1L is a mollusk-specific gene^51^, and a novel Dmrt gene (EsDmrt-like) was identified in *Eriocheir sinensis*^52^. These results indicate that there may be more members of the Dmrt gene family, especially in aquatic invertebrates. Therefore, it is necessary to conduct systematic identification and analysis of the Dmrt gene family in other classes of invertebrates.

## Conclusion

In this study, a systematic analysis of Dmrt family genes in 11 representative echinoderms was performed. A total of 43 Dmrt genes have been found, and the number of Dmrt genes in different echinoderms ranges from 2 to 5. The phylogenetic tree showed that all Dmrts from echinoderms were classified into 5 classes: the Dmrt2-like class, Dmrt3-like class, Dmrt4/5-like class, Dsx-like class, and novel Dmrt class. Furthermore, selective pressure assessment suggested that the Dmrt genes underwent purifying selection pressure. In general, this study provides a molecular basis for echinoderm Dmrt and may serve as a reference for in-depth phylogenomics.

## Supplementary Information

Supplementary Information.

## Data Availability

The datasets used and/or analyzed during the current study are available from the corresponding author on reasonable request.
