# Supplementary material for: Genome-wide identification and comparative analysis of Dmrt genes in echinoderms
Source: Sci Rep. 2023 May 11;13:7664. doi: 10.1038/s41598-023-34819-z (PMC10175285; doi:10.1038/s41598-023-34819-z)
Supplement: Supplementary file 1 — Supplementary Information. [file 41598_2023_34819_MOESM1_ESM.docx]

Table S1 Accession numbers of the known Dmrt sequences

| *Species* | Gene ID | Proein Accession Number |
| --- | --- | --- |
| *Homo sapiens* | HsDmrt1 | NP_068770.2 |
|  | HsDmrt2 | NP_870987.2 |
|  | HsDmrt3 | NP_067063.1 |
|  | HsDmrtA1 | NP_071443.2 |
|  | HsDmrtA2 | NP_115486.1 |
|  | HsDmrtB1 | NP_149056.1 |
|  | HsDmrtC2 | NP_001035373.1 |
|  | HsDmrtC1 | NP_001074320.1 |
| *Mus musculus* | MmDmrt1 | NP_056641.2 |
|  | MmDmrt2 | NP_665830.1 |
|  | MmDmrt3 | NP_796334.2 |
|  | MmDmrtA1 | NP_783578.1 |
|  | MmDmrtA2 | NP_758500.2 |
|  | MmDmrtB1 | NP_063925.1 |
|  | MmDmrtC2 | NP_082008.1 |
|  | MmDmrtC1 | NP_081867.1 |
| *Macaca fascicularis* | MfDmrtC2 | XP_005589481.1 |
|  | MfDmrtB1 | XP_015291127.2 |
|  | MfDmrtA2 | XP_005543469.2 |
|  | MfDmrtA1 | XP_005581667.2 |
|  | MfDmrtC1 | XP_015299833.1 |
|  | MfDmrt2 | XP_045229108.1 |
|  | MfDmrt3 | XP_005581900.2 |
|  | MfDmrt1 | XP_005581901.1 |
| *Bos taurus* | BtDmrt3 | XP_002689673.4 |
|  | BtDmrt2 | NP_001179302.1 |
|  | BtDmrtB1 | XP_024845606.1 |
|  | BtDmrtA1 | NP_001179569.1 |
|  | BtDmrtA2 | NP_001096751.1 |
|  | BtDmrtC2 | NP_001033271.1 |
|  | BtDmrt1 | NP_001071528.1 |
| *Balaenoptera musculus* | BmuDmrtC2 | XP_036690564.1 |
|  | BmuDmrt1 | XP_036711655.1 |
|  | BmuDmrtB1 | XP_036712433.1 |
|  | BmuDmrt3 | XP_036712559.1 |
|  | BmuDmrt2 | XP_036712720.1 |
|  | BmuDmrtA1 | XP_036712737.1 |
|  | BmuDmrtA2 | XP_036726057.1 |
| *Gallus gallus* | GgDmrt2 | XP_003643035.3 |
|  | GgDmrtA2 | XP_015146712.1 |
|  | GgDmrtB1 | NP_001232910.1 |
|  | GgDmrt3 | XP_429193.2 |
|  | GgDmrt1 | NP_001095301.2 |
| *Oryzias latipes* | OlDmrt1 | NP_001098150.2 |
|  | OlDmrt2A | XP_023813898.1 |
|  | OlDmrt2B | XP_004068077.2 |
|  | OlDmrt3 | XP_023813900.1 |
|  | OlDmrtA1 | XP_004079711.1 |
|  | OlDmrtA2 | XP_023810346.1 |
|  | OlDmrt1Y | NP_001295953.1 |
| *Oreochromis niloticus* | OnDmrt1 | XP_013126366.1 |
|  | OnDmrt3 | XP_003444527.2 |
|  | OnDmrt2A | NP_001266696.1 |
|  | OnDmrtA2 | XP_005479065.1 |
|  | OnDmrt2B | XP_005457139.1 |
| *Larimichthys crocea* | LcDmrt1 | XP_027133892.1 |
|  | LcDmrt2A | XP_010734871.2 |
|  | LcDmrt2B | XP_019120792.1 |
|  | LcDmrt3 | XP_010753534.3 |
|  | LcDmrtA1 | XP_010735509.1 |
|  | LcDmrtA2 | XP_027145940.1 |
|  | LcDmrtB1 | XP_019113286.1 |
| *Drosophila melanogaster* | DmDmrt11E | NP_511146.2 |
|  | DmDmrt93B | NP_524428.1 |
|  | DmDmrt99B | NP_524549.1 |
|  | DmDSX | NP_731197.1 |
| *Bombyx mori* | BmDmrtA2 | XP_021202446.1 |
|  | BmDmrt2 | XP_004930266.1 |
|  | BmDSX | NP_001104815.1 |
| *Aedes aegypti* | AaDSX | NP_001345310.1 |
|  | AaDmrt2 | XP_021707667.1 |
|  | AaDmrtA2 | XP_001649612.2 |
|  | AaDmrt3 | XP_021702532.1 |
| *Sagmariasus verreauxi* | SvDSX | ARK36621.1 |
| *Cherax quadricarinatus* | CqDSX | QDM55346.1 |

Table S2 The genome database information for the studied echinoderms

| **Species** | **Database** | **Website** |
| --- | --- | --- |
| *Acanthaster planci* | NCBI | https://www.ncbi.nlm.nih.gov/genome/ |
| *Anneissia japonica* | NCBI | https://www.ncbi.nlm.nih.gov/genome/ |
| *Apostichopus japonicus* | NCBI | https://www.ncbi.nlm.nih.gov/genome/ |
| *Asterias rubens* | NCBI | https://www.ncbi.nlm.nih.gov/genome/ |
| *Hemicentrotus pulcherrimus* | HpBase | https://cell-innovation.nig.ac.jp/cgi-bin/Hpul_public/Hpul_annot_home.cgi |
| *Holothuria glaberrima* | Ryan Lab Genomes database | http://ryanlab.whitney.ufl.edu/genomes/Holothuria_glaberrima/ |
| *Lytechinus variegatus* | NCBI | https://www.ncbi.nlm.nih.gov/genome/ |
| *Patiria miniata* | NCBI | https://www.ncbi.nlm.nih.gov/genome/ |
| *Plazaster borealis* | GigaScience database | http://www.gigadb.org/ |
| *Strongylocentrotus purpuratus* | NCBI | https://www.ncbi.nlm.nih.gov/genome/ |
| *Temnopleurus reevesii* | TrBase | https://cell-innovation.nig.ac.jp/Tree/ |

Table S3 The amino acid sequences of the identified Dmrt genes

| Gene ID | Sequence |
| --- | --- |
| Tr-TRE_26769 | MLRDTKSLMNHHAAQDMDRIDIRTLSLLRGPLTEKGARKPKCARCRNHGMISWLKGHKRHCRFRDCRCAKCNLIAERQRVMAAQVALKRQQAAEDAIILDNLLNGENRNS |
| Tr-TRE_05017 | MNFDVITELSKVPCQKATPKHQAASRRVVRTPKCARCRNHGVVSCLKGHKRFCRWRDCRCTNCLLVVERQRVMAAQVALRRQQSIDSTSGNGGRKPPGSLAAPGGGDSGEGKKTTKEISAEELSSSKRKLAEIGAEASRLKERVKRLSSTAARGRINGSIARDILEGYRNQSGRLPSRLPNRPVIFLPPPVSERMRKRRAFADKELETTMLQRECQWTLMLAYAAGDKSLAPFASSNPAMHLDASQLGTPNDHLRTLDKRLEHLTHAHRPLVKGADVMWNGIADGDDCLRRGGGKMPKLSPRVVEDVTASYAAADSTAISEIPAVFYSNVHRLNRLPEIYDGSSKQQSALRNDTLEDSPSTKSENVYLKFSSTVADAEDDRRTGTETSCNLPLSGDPHIYGIDQLPKNSSSPCHVGRECASLKHNVNKNEHELNERNKAGSSSNGGTRCASKNHLRFSIESLLRK |
| Tr-TRE_16693 | MALDEMGINPNNHCRMMGIFIFSHGYSEAIEIENEGDGKEEDDELQRCLPLKKRHPTCARCRNHGLILDLKGHKRHCEYQDCRCSKCILVTQRRLVMAKQDALSREQIRKQRPDREATLQMQNGRQFYKEIQQSPPTSAVDHTTEGHRLKEMSHPVVQPSPDSTNMSRFDLINPNTLKYFKSMSPLLVSHHNFKCTRITIRAQKGVIEDKKLAILSERRLLYCSNSQYSPWDSSPQPTQISVSPETISEPQISQHYPASFHSARRDKTYHPGMREDEIYRPGMGFIANIMANPDIAPLPSQSCTTIDSHPSEQVMGGVRNVNIPSAQSSFRQASGFRRSSSLRSLQADESYRANALTVPQDLPNSMVSYASINGPAGSFQSRFAPTLVNHSAPRSTFIAKPPVQPIVSNPILPHDSIDTMSRYDPSRSSAPSMNLSAPCSFHSRVSQCYSTSIDPIPSPGDQPHHHPNVHSEHLANVGQFKGQTVPMDSYTGSIRTAQRTSISPDQHPQQFSTYLVNEFQTKAKPWNTMSESPYQDSPQYYSHVARAFQQPYYIPSPQSDNCFVQPQGEGRIEPPPIIFPAHSDDSSVQPIESYSTISSQDTEDTSSNETEDSSTEEDTSSPDIPQEPCDEPSYKSRRPFPLRITIPSLLSNQSSNTSNLSPMDPSPTFSPASLSLLSHITLPTNDFPSPGFFTSSLPSFNYPSPTLPTQTSPVFSYPSPYTNPSKHSKTTPFPDLPDTPMPVVEPNQPMSAFIEQVRIPPQNDEDQDDQEDAEIDVCTLDHYACQLSIVRMYL |
| Sp-XP_786938.3 | MNLGGPGDPPAHTHPPHTSTSPNDMPVVPPQLQPGHHIHRPAHHHLHQSGMPPPPGGPTGMLLRAEKPYPRTPKCARCRNHGVVSALKGHKRYCRWRDCICAKCTLIAERQRVMAAQVALRRQQAQEENEAKELGLYYETSDGAIYAMNGIAVQTHKGYDPYRNQQSEQGQGQGQGQAQQGGGADQKRARLEIIRAPASPPSANPAPPHSPTSLPNQDPRVSSPDTRSPRSVSAGTMSPTKSLSPVASPRIESAEQSEVIRTPGFGMIQPGSGLDFEHSEARRLGFMQQQPSTTASAAAALASSLSFAGQSGLGGGKGQRPPIEVLCRLFPTQKRAVLELMLQGCDGDVVQAIEQLLNCQREVSASSSTSSTPSTALTTDSVTYPTTATTVPSVAEPPCIAHRPYLSTTPVCTAGLKSAFSPLTASHDKTLIQPPVTHAGLPPMRLAYPSYPRGITFWNPYTSAMIPAAFGVQQPAECHFNGIMGGPRKDNTRPNGAFGGGSP |
| Sp-XP_030852253.1 | MLRDTKSLMNHHQDMDRIDIRTLSLLRGPLTEKGARKPKCARCRNHGMISWLKGHKRHCRFRDCRCAKCNLIAERQRVMAAQVALKRQQAAEDAIILGLRACSPSGPYNYLPQGPIFGAAPPGSGDGCNADEDDDDDDDDMGQTCDSPLDDVDMPPSPERETTEALKEIKDDIERESPTTSVPEPPKKVVEKHELPEPSEKPNEDGEEPKKKAKLSGPLDTFNGMASSFVSPFRPGRLSQTEILTRLFPHQRKAVLELVLQGCSGDLVKAIEHFLSAGESVKNNGSASSRSDHAHSSEKDQNEMYHIPSGLPTIPGLGSSIFRTPMHTDKLGVGGMKSAFTPLPPASSAPLPLLFSHRPPNPFQADALLGRTSIFAPSSLSDLHVGAAGPGRFVFPAMHPLNLSGKLAAAASEGYPRYVFAPYPTCPPDCTQYPSLHSTRAPAGSTGSDSEKSPGAIADLSVTSNVDSD |
| Sp-XP_030851643.1 | MPGHKVLPKHQPSVRRVVRTPKCARCRNHGVVSCLKGHKRFCRWRDCRCTNCLLVVERQRVMAAQVALRRQQSSDQGSGNGAGKAPGSDTASGGRGGVDGRKRSAKDVSPEELSNSKRKLAEIGAEASRLKERVKRLSSTAARGRFHGSIARDILEGHRGKPGRLSSRVPNRPVIFLPPLVSERMRKRRAFADKELETTMLQRECQWTLMLAYAAGDKTLAPFASNPVSYFDVGRTGNQDAHKGHLGAMGTRLENLNQARETMTKGSEASWKGSLGGDERLRCGVGEVPTLSPGVAERVAASYMRTMNPLMMSSVPQTPFKQDHRVGPGQFPVNSYPHSAQTTVVMNEKPYDRTREDTYLHASKALTLSKGERRTGDESLSSSSQDGTELDNRPEDVEYVIPRSGDSDADLGCVSPDHDGNRNGQELGQKGGEGPSGAPGVVVVQSSYETKRQAPKSYLAFSIESLLKK |
| Sp-XP_030854341.1 | MAASPIKVWILLQYICYFYYPLPLLDEEDGIGLHVDDELQRCLPLKKRHPTCARCRNHGLILDLKGHKRLCEYRYCRCTRCIVVSQRRVVMAKQVALSREQVRQHRQDQDTSEQLRESQRFHHDVPLPPPVIDLTSEGPNSDEMAYPVVQPSPDSTNIGYGNSPYSSWDSSQANLSQPTQMSSSSSQNVFGQASQYYPTSFQSAQKLDSHRPEMFFTANPSSRRIMPSPIMPYQSSDPRVTNLSDRIPGASPASQGNFEQAPHYHSASFHTSQVDEMYRPQVSLSANRTVRPLTPSSMVTSYESMDPPSGYFPSRMTATSANLSSAHSVMGQASQYCPRPSFHSSLVDETYQCQVSVTANYKTGPVFQNPMMDTESAYRDSYMSANPQMNSTAAPRISSMPNTFFRAGETVQSMCYCNVPDQAQFHPQVPTFPSSLRVSPFDEQATPMSSLPPSVVTAAQRSYINTDRHPSPRVTSTAAYTFHTIDEPPFYSQNDIPYQDDEPAFRSQRATTSEQSQDMPLDHSDDFSWSPQAVTATKSPEAVSSDHSPEPSYKSRRPFPLRITIPSLQTNESSFTDIRSPIDPSPTFSPFSLSLLANTTLPTSDIPSPGFFTSSLPSFNYPSPTLPTQSSPVFSYPSPYTNPSKYAGVPSFQSLADSLLPVMQTSQVTSNFAEQVRIPPKYDEDLDDEREGDEIDVCSLGHYASDNF |
| Sp-XP_030839690.1 | MAASPIKVWILLQYICYFYYPLPLLDEEDGIGLHVDDELQRCLPLKKRHPTCARCRNHGLILDLKGHKRLCEYRYCRCTRCIVVSQRRVVMAKQVALSREQVRQHRQDQDTSEQLRESQRFHHDVPLPPPVIDLTSEGPNSDEMAYPVVQPSPDSTNIGYGNSPYSSWDSSQANLSQPTQMSSSSSQNVFGQASQYYPTSFQSAQKLDSHRPGMVFTANPSSRRIMPSPIMTYQSSDPRVTNLSDRIPGASPASQGNFEQAPHYHSASFHTSQVDEMYRPQVSLSAYRTVRPLTPSSMVASYESMDPPSGYFPSRMTATSANLSSAHSVMGQASQYCPRPSFHSSLVDETYRCQVSVTANYKTGPVFQNPMMDTESAYRDSYMSANPQMNSTAAPRISSMPNTFFRAGETVQSMCYCNVPDQAQFHPQVSTFPSSLRVSPFDEQATPMSSLPPSVVTAAQRSYINTDRHPSPRVTSTAAYTFHTIDEPPFYSQNDIPYQDDEPAFRSQRATTSEQSQDMPLDHSDDFSWSPQAVTATKSPEAVSSDHSPEPSYKSRRPFPLRITIPSLQTNESSFTDIRSPIDPSPTFSPFSLSLLANTTLPTSDIPSPGFFTSSLPSFNYPSPTLPTQSSPVFSYPSPYTNPSKYAGVPSFQSLADSLLPVMQTSQVTSNFAEQVRIPPKYDEDLDDDREGDEIDVCSLGHYASDNF |
| Lv-XP_041474728.1 | MNLGGPSDPPTHSHPPHTSTSPTDMPVVPPQLQPGHHIHRPAHHHLHQSGMPPPSGGSTGMLLRAEKPYPRTPKCARCRNHGVVSALKGHKRYCRWRDCICAKCTLIAERQRVMAAQVALRRQQAQEENEAKELGLYYETSDGAIYAMNGIAVQTHKGYDPYRNQQQSEQGQGQTQQGRSNNSVFFFTFSRSTWLKLMWNLLIANVFVEGISIPDDKKMNIKTSVALLTI |
| Lv-XP_041474858.1 | MAEGKETNVTTRKISESLNTEIISDPSLKKNMTSGLTTERNKHLDKKSLPKHQASVRRVVRTPKCARCRNHGVVSCLKGHKRFCRWRDCRCTNCLLVVERQRVMAAQVALRRQQSNDPSASGNGAVKTPGSGSASGGEREGGNVNGCRKKSAKELVSAEELSDSKRKLAEIGAEASRLKERVKRLSSTAARGRINGSIARDILEGQRGKPGRLSSRVPSRPVIFLPPPVSERMRKRRAFADKELETTMLQRECQWTLMLAYAAGDKTLAPFASHPFSSHFDAGRHTGQDTDKEPLRNIRTGYGGLNHVSGETRSNESELLWTGSPCNDVRLQLGGGGEVPTLSPSVAVPCMRTPNASMVSIPSTLIKHGHGAGVGLGQFSGNLHRNPALTTIVTNKEHRERSHEDTYLHDPAKLPLSRCKRLKGDERLGSSSQNSRFGGEFDSRPEEADFRGSGSMDRDTEHAKWRVVGRDHDMHGHGKQLGQDSAEIGRDGSEFEAQDFCEKTIQASTNNLAFSIESLLKK |
| Lv-XP_041475654.1 | MLRDTKSLMNHHHQDMDRIDIRTLSLLRGPLTEKGARKPKCARCRNHGMISWLKGHKRHCRFRDCRCAKCNLIAERQRVMAAQVALKRQQAAEDAIILGLRACSPSGPYNYLPQGPIFGNAPPGSTDGGNVDEDDDDDDDDMGQTSDSPLDDVELPPSPEHEKTEVMREMKEDIERESPTTSVPELPKKIVEKHEPPEPSEKPTEDGEEPKKKAKTSGPLDTFNGMASSFVSPFRPGRLSPTEILTRLFPHQRKAVLELVLQGCSGDLVKAIEHFLSAGESAKNNGSASSRSEHASHSSEKDQNEAYHIPSGLPTVPGLGSSIFRTPMHADKLGVGSMKSAFTPLPPASSAPLPLLFSHRPPNPFQADALLGRTPIFAPSSLSDLHVGAAGPGRFVFPAMHPLNLSGKLAAAASEGYPRYVFAPYGTCPPDYTQYPSLHSTRAPAGSTGSDSEKSPGAIADLSVTSNVDSD |
| Lv-XP_041467722.1 | MDNDGADVKGREDESQRCLPLKKRHPTCARCRNHGLVVDLKGHKHLCEYRDCLCTRCNLVIQRRVVMAKQVALSREQLKQQRQDHCTSQQLQVSQGSSQGIPSSPTVIDLISEGTRSEERANSVVQLSPDSTNVEYNGPYSSWDSPPMNLSQPTQMTLTPQNVSSQASRSSPTSFQAVQESETFRPGMVFFTSPSNRSLHPVVPYQSSAPRIVSNNDNIPGASAFINYASSQGNFDHALEYSPTSYHTPQLNEMYRPQPLVGANQMPRPFVPSPAIQMASNESINQHIGYIPSQTATYSANLSSAHGVTGQTSEYPANHLFAHGVTGQIAASSANHLLAEGVSGQASEYSANHMLAHSVTGQTTAYTANHLLAHGVTGQTAAYSANHLAQGVTGQASEYFANHSSAHGVSREIEFHPRSTFHSSLVDETYRCQVSITSNYPTGPAFHCPVVPHQPFDAVSAYRDSYMLANSRVASSSAMHIDSMSETQPPSEEATDLSNIPYCQVKEHADLLHQTPLSSSLRVSSFVNQSAQNFMPAQEYYSNPDRYPSCSLPADSATAAYLFQTGGRAMLSSESDNSFQGADFTRRPQIDTTPLQPEDTPLNHADFSSRSVPLTTTQQEIAVNISKEPTYKSRRPFPLRITIPSLNLSDRFNDSLSPSPMDPSPTFTPSNLSILTNTSLPTSDIPSPGFFTASLPSFNYPSPTMPTQSSPVFSYPSPYTNPSKHAGLPSFQSLTESILPVTQSSLLPSNFAEKVRIPPKYDEDFENDYESDEIDVCSLNHYAQ |
| Hp-HPU_09801 | MLRDTKSLMNHHQDMDRIDIRTLSLLRGPLTEKGARKPKCARCRNHGMISWLKGHKRHCRFRDCRCAKCNLIAERQRVMAAQVALKRQQAAEDAIILGLRACSPSGPYNYLPQGPIFGAAPPGSGDGCNADEDDDDDDDDMGQTCDSPLDEVDMPPSPGETARAPLTPTDVGADIGNDCERETTEALKELKEDIERESPTTSVPEPPKKIVEKHEPPEPSEKPNEDGEEPKKKAKLSGPLDTFNGMASNFVSPFRPGRLSQTEILTRLFPHQRKAVLELVLQGCSGDLVKAIEHFLSAGESVKNNGSASSRSDHAHSSEKDQNEMYHIPSGLPTIPGLGSSIFRSPMHTDKLGVGGMKSAFTPLPPASSAPLPLLFSHRPPNPFQADALLGRTSIFAPSNLSDLHVGAAGPGRFVFPAMHPLNLSGKLAAAASEGYPRYVFAPYPTCPPDCTQYPSLHSTRAPAGSTGSDSEKSPGAIADLSVTSNVDSD |
| Hp-HPU_09660 | FNMFEVLYAIVHRDRLTMADGSDIVIVTSIETNETVNTEIGAYPSPKKTIIFDLTTDRNKTQMPNHKVLPKHQPSVRRVVRTPKCARCRNHGVVSCLKGHKRFCRWRDCRCTNCLLVVERQRVMAAQVALRRQQSSDPGSGSGAGKAPGSDTASDGRGGGDGRKRSAKDVSPEELSNSKRKLAEIGAEASRLKERVKRLSSTAARGRLHGSIARDILEGHRGKPGRLPSRVPNRPVIFLPPLVSERMRKRRAFADKELETTMLQRECQWTLMLAYAAGDKTLAPFASNPVSYFDVGRPGSQETQKGQLGSMGNRLENLNHARETMTKGSEALWKGSLGGDECLRCSVGEVPALSPGVAERVAASYMRTMNPLMMSSVPQTPFKQDHRVGQGQFPVNSYPHSAQTTVVMNERPHERTRENAYLHASKALTLSKGERRRGDESLSSSWQDGKCGIELNNSPEEVEYVNPRSVDSDADLGCVSPDHDGNTSGQELGQGGGEGPSDGPTGVVVVQDSYETKSQAPKSYLAFSIESLLRK |
| Hp-HPU_07004 | SWKQRLRKHSDARKRHPSQTKLKVQDDHEANGIHDGLDSHSIHLLFYYLPILDEEDGIGLDDELQRCLPLKKRHPTCARCRNHGLILDLKGHKRLCEYRDCRCTRCNLVSQRRVVMAKQVALSREQVRQHRQDQDTSEQLQKNQRFHHDVPLPPPVIDLTSEEPNLEEMAYPLVQPSPDSTNIEYGNTPYSSWDSSQSNLSQPTQIMSSPSQNNFGQASQYYPTSIQSAQKLDSHRPGMIFTANPWRGQTIPNPMVSYQSSDPRVTNLSDQIPGASPSSQGNFEQAPHYHSASFYTSQADEMYRSQVSLSANRTTRPLTPRPMVSYESMDPPAGYFPGRMTATSANHSSAHSVMGQASQYCPRSSFHSSLVDETYRCQVSVTANYKTGLIFQSPMMDTESAYRDSYMTVNPQVNSAAAPRTNSMPNTSFRAGDTVQSMCYCNVADQAQFHPKVSTFPSSLRVSPFEEQAAPMSSLPPSVVTEQRSYITPDRHPSPRVTPTAAYTFQTTDEPPFYSQNDIPYQDDEPTFRSQIVTTSQQPKDMPLVRSEDFSWSPQAVTAAKNPEAVSSNHSHTPSYKSRRPFPLRITIPSLQTNESTFTDIRSPIDPSPTFSPFSLSLLANTTLPTSDIPSPGFFTSSLPSFNYPSPTLPTQSSPVFSYPSPYTNPSKYAGIPSFQNLADSLLPVMQTSQVTSNFAEQVRIPPKYDEYLDDDREGDEIDVCSLGHYASDNF |
| Hg-Hglab.06749g1.t1 | MSLDNTFHNRPVSSTDMPVVPNHPGPAHHIPHPQHSTASHQPTSTPMLLRADKPYPRTPKCARCRNHGVVSALKGHKRYCRWRDCVCAKCTLIAERQRVMAAQVALRRQQAQEETEAKELGLYYETSDGAIYAMNGIAVQTHKPYDPYRHQEGPGELPYFLHQVA |
| Hg-Hglab.14937g1.t1 | MFSSVDRSPMDIRTLSLLRGPLTEKGARKPKCARCRNHGVISWLKGHKRHCRFRDCRCPKCNLIAERQRVMAAQVALKRQQAAEDAIIMGLRACSPDGHYGYLPPGPMFGSRGDVEGDDIDKESIDAGDTENQDLTGEKSIQNC* |
| Hg-Hglab.00147g3.t1 | MRDYSSCAIELWRRDKVSYLVLLSPGKELYGTGGFLASWNLPGRVNMAMDLSKNISLKATPAVTGKSSSGTRRYARTPKCARCRNHGVVSCLKGHKRLCRWRDCRCTHCLLVAERQRVMAAQVALRRQQTNSSQNGNKTDTKSGANKEQSDGDEKKNRGNAATLGEKELKERLLSKSKLEKSIAEDVLKRTSGPRRYLRRFPPTSMSRPPILFPPGVSERMRKRRAFADQQLEAIMWQRECQQACTMIGGQRTTLPPSDSSRRTVSEQTDVPKKSDIFRLTSGNLTQTNLNQTGNSEPHRGTKPLSTSSTNFAASVHNIKLMNTVMPRPFVLYLSNYIPIPPSLITNVGTVPSSLATSFLENRSSTTIPIQDETSSSSGSPLSYDDDTVSPVESPIGEPNHTRNTDFSIKNLLES* |
| Hg-Hglab.05111g1.t1 | MEVAMEPRASMCKTVPKCAQCRNHNQDVPRKGHKGRCPWSKCTCTKCIVTFEKRQLAATRQRMRREPLEDGESEQQDEVQLLLNLKSTIEKKEKKKGSHETASEGKQKYRYSGNIQNNMYFPANLQPEDTVKPKDSKGVSNLPPGDVVTPSGYNTDLSWPPPCPFCPCNHWEMVLPPTQQVPMFNPEGCQYMPHQLPNAWASPYNHYLEHGFHNQESFVTRFPAPSESQPLESNSRFQYQPFTTAQYPFNNGPLNLQNNNPRFASSDHTD* |
| Ar-XP_033636556.1 | MSLSGGHIDSQQNSDMPVSAHHSNARHHHPTLLIRTEKPYPRTPKCARCRNHGVVSALKGHKRYCRWRDCVCAKCTLIAERQRVMAAQVALRRQQAQEENEAKELGLYYETTDGAIYAMNGVAVQTHKPYDQYHRSGSPESKRPRLEIIRAPTSPTASSTSALSPPNPISPNLSVEPHTRADTETITQRHMDERVSPPMKLTSSPTPSPRGGDMPLSPRSDGSGRMLREDYEREGYREQNSAGFMEQLSPAQLLSTFHSRKGPQRAPVDVLCRLFPAQKRSVLQLVLQGCSGDITQAIEQILNNQREEASSGPVTTSMPTPCSPVTASLPASFPPSATVTTDSHGAYIAHRPYLPNSQHLNPGGMKSAFSPLTTAMPMPNMDKAPSSVVASNIPQMRFAYPPYPRGLTLWNPYQPTVIPTAFGVRPATAAEYTFSGIMRDLSNGHGGKDCRPNGAFGVGATCD |
| Ar-XP_033636851.1 | MTTPRTQTTRKVLRTPKCARCRNHGVVSCLKGHKRYCRWRDCQCTNCQLVVERQRVMAAQVALRRHQATEGSSPVSGKDGQPIGSGPPRKVPCGTGKGTGGSGARKTAPLRSVGSGSTSVSKDILDGCRSRSSRSSHATASTSPRPVIFLPPSVSERMRKRRAFADKDLESTMLQRECQWSLMYAAQAGLSRLQPPGTEHHRRASTDTTTPLTDQHGDITSQTKHFLRRLFPSLSTSTLDAALRCSGGNLRLAIEKLVTVHSPPSQLGPGTRPEAEHGVVPESTLYLPWYLSYQRNASLLKENDLRTHTTTPFEQRDTNSLLASRTIPKYSMQEFRGDSSVSAFTSLQREGNSIPFSDSRNRFGSVESECEYSRDRDEESILTNSQDACGPNQTHSKRFLKTPSTSRLSFSVDSIMGKR |
| Ar-XP_033637002.1 | MMMERPVIDLQTLSLLRGPLTEKGARKPKCARCRNHGVISWLKGHKRHCRFRDCRCPKCNLIAERQRVMAAQVALKRQQAAEDAIAMGLRACSPGSGGGFGMMSANGPMFGSSDSGEELSPRPMDIADEEADHHDIPEKLVKNESSNEKENGHRTPSSGKPEHNNNNSNSSSKNKSDSLDTFNGFAASFVSNFRPGRLTPIEILTRLFPTQRKAVLELVLQGCSGDLVKAIEHFLSASETQHSPNAASIPSSISRAGERPSIGHHDSFGMSGVHGLQTAFRSSLSSEKHPVGSMKSAFTPLPPSSASAASSVPLLFAPRPPHPFHAEALLGRTPLFAPTSVAELTSSMPSPAMGSPRFAFPALHPFHLSGKFPSAGEGYPRLIFAPYATCPPDCVQCPGSRPGMHSEPEKSPSGTTDGSVANSAVDSE |
| Ar-XP_033629679.1 | MSLSSQCKKKCYTSMIKRNVRELEIEVLHEFLVAKMNGEGERTVRTQSSSSTRSSRKLRTAAAPSVSTVPPVTAQADASADASPPEFDGQGLRKKRAPRCSRCRNHGYLVPLKGHKKYCRFKLCKCSYCQLITDRQRVMASQVALKRQQDMEEVVAGCRNATGVVALPPLKEHLTFKSSCTATSQSLHTVHSGACNNQGNTQLHNYSPNTHPTFQPQWQNNSSTTPIPPDLTCSVENHTFSVVDPAETQSSTFGPGFPHGGSFSSMTPFAVYNDSHGSSFPLMFNAAKFPPNQMFSSEYLAYQRQLHLQHQMQQQRLRDNSMGNEFGYCNGIQGAFIPLNHQSSPSGADDSLDEDILEIATDEGPGGTDDGLGHACPASTNIDRTHVTQRTTLHGFPSPPATMSDFHSLPVNVSVTQAKTFIPLQHALFPQGQSQDKKCLL |
| Apj-PIK34621.1 | MDMRTLSLLRGPLTEKGARKPKCARCRNHGVISWLKGHKRHCRFRDCRCPKCNLIAERQRVMAAQVALKRQQAAEDAIIMGLRACSPDGHYGYLPQGPIFGSRGETEADDLDKDSLDGGDTDIQDVADNNEITTVTVDDRKQDSDRESPTTSVPEPKTNLTTSEKADDSMKRPAENKTTENSPSPDKKIKKSSTLDTFNGLAASFVSPFRPGRLSR |
| Apj-PIK44057.1 | MDMRTLSLLRGPLTEKGARKPKCARCRNHGVISWLKGHKRHCRFRDCRCPKCNLIAERQRVMAAQVALKRQQAAEDAIIMGLRACSPDGHYGYLPQGPIFGSRGETEADDLDKDSLDGGDTDIQDVADNNEITTVTVDDRKQDSDRESPTTSVPEPKTNLTTSEKADDSMKRPAETKPQRTALAEDRNKEIVDIRYIQRSRSELRRPPHPFQHESLLGRAPVFASSALGDLSTSGASAPGRFVFPALHPFSLAGKLPNSADMFPRYLFAPYASCPPECVQCPGLIRGGHGTEPSSPSEDTPDGTVTSNTKNESD |
| Apj-PIK33706.1 | MVSLRSHRYTSAAVLNWGLFCVFDVGCIMSLENTFHNRPVSSTDMPVVPNHPGPTHHIPHPQHSSASHQPNSTPMLLRADKPYPRTPKCARCRNHGVVSALKGHKRYCRWRDCVCAKCTLIAGAEGHGCQVALRRNRPGRDGSQGIRTLLRNIRRSYLCNEWNCCANT |
| Apj-PIK41536.1 | MDLECLGPMARLRSEPKCAKCRNHHEIVPRKGHKNRCPWFNCYCKKCSSTTEKREYFARRQKYKRDLKLSSPDQNINEDVQLLLNLKVNNTDKKVKPPDSIEEPTESKWRTPDYKWKYRFSGTIQNNMFYPAVPNTSAVQPHAVMSPTKQFEAVSPPEESPQSPKVSKVKACSESCPYCTCSHIEKLYPPTQQKQSYTDLEDYQPTSHEPPGPLMSTPNYYPEQWSPEPDIYCEPYQTHYLDSVSQVTVREQPHHSPTPSLRYPFNANVVSQNMINFRFMQR |
| Apj-PIK43860.1 | MSVSSLQGHKRLCRWRDCRCTHCLLVAERQRVMAAQVALRRQQTSNSAQNDKNSNKNSNKHIHSDRHKLQDNGKEEDIKPSDTSKEEEQLRERLISKTKLEKSIAEDVLKRTSGARRYLRRFPASTISRPPILLPPGVSERMRKRRAFADQELEAVMWQRECQQACTMIGSRRINFPPQTDSSRLVSHPREETELTRRPNILNTPPQRNASQIDSSTENQLNLSVDSALSPTCSNFVANLDQLKFINPTLQRPLFLYLSRYVPLPVPDLLRLRTARGNVTSFERNCVHGEVPPLSEQTSPLPDSPSSCGDESVSSPESAIAELSCTTTGRKTDFSIRNLLEN |
| Ap-XP_022089262.1 | MNLSGGSIEPQQNSDLPASAHHPPTSLHHHHPPPTLLLRAEKPYPRTPKCARCRNHGVVSALKGHKRYCRWRDCVCAKCTLIAERQRVMAAQVALRRQQAQEENEAKELGLYYETSDGAIYAMNGVAVQTHKPYDQYHGSTSPESKRPRLEIIRAPRSPSASCGSAMSPPNPTSPSLTVEPHPRTDTETVSQRHVEERVSPSMRLTSPTPSPRGDVPSSPRSEGSGRMLREEYGDRDAYREHNGGDFADQLTPAQLLSFHGRKGPQRPPVDVLCRVFPAQKRSVLQLVLQGCNGDITQAIEQLLNNQKDESSAVSMPASLPVTGTPVTAALQANFPPTATVTTDSHGAYITHRPYLPNNHHMNPGGMKSAFSPLTTAMPMPSVDKTPPTVVASNIPQMRFAYPPYPRGLTLWNPYPPSVIPAFGVRPATAADYTFSGIMRDLSNGHSGKECRPNGAFGVGATCD |
| Ap-XP_022087749.1 | MDNYMVPNLTLHVHQDESHLGISSPSPTSAEADSRSGPRLKAGKMDPPSRPPTTRKVLRTPKCARCRNHGVVSCLKGHKRYCRWRDCQCTNCQLVVERQRVMAAQVALRRHQATAEDAPVAPGKEAAGNGVVAPTRKSVCGAGKGVNTSGGSRKIHSSRGQGHRATSVSKDILEGCRSKGSRTTAPSTSVSARPVIFLPPSVSERMRKRRAFADKDLETTMLQRECQWSLMYAAQASLSRLPSSVNRQYHPHHRTPGGIDAVREPPDHTAGFLRRLFPSYSTTALDAALRGSAGNLRLAIEKLVTAYAATPTCGPTAHPLLSTGVNLETTPSLPWNLSPPGSKIYEEDDGRTNSAHFEWGSILPVNKPIQKFSINRIRGEGSVSAFSSVRRAENWASDSRSRLGSVESENDYSQDLSADLAPAGDRKRDRLDDSNAQEICRNASLTKRFAKTVSTKRLSFSVDSIMGKS |
| Ap-XP_022089263.1 | MMERPVIDMQTMSLLRGPLTEKGARKPKCARCRNHGVISWLKGHKRHCRFRDCRCPKCNLIAERQRVMAAQVALKRQQAAEDAIAMGLRACSPGTGGSFGMMSANGPLFGSSDSGDELSPRPMDIVDEDGDHQDITEKKTKIESANEKENGRGTPPSSQSERNNNNRHSSGKSKSESLDTFNGFAASFVSNFRPGRLTPIEILTRLFPAQRKAVLELVLQGCNGDLVKAIEHFLSASEDTHQHSPSGAALPPPPPISRGVVGERPSVVGHESFGQSGVHGLQAAFRSTLCSEKHPVGSMKSAFTPLPPSSAAGSSVPLLFTPRPPHPFHAEALLGRTPLFAPSSVGDLSSSMSSPAMASPRFAFPALHPFHLSGKFPSAGEGYPRLIFAPYATCPPDCIQCPGSRTSMHSEPEKSPSGTTDGSIANSAVDSE |
| Ap-XP_022111791.1 | MGKSIKKRHGSKRATEKRRCCEDQGEVVESTSDSPIGPGRDLARNVKGAGEAPTSPGIHGDGAVERGPVTGSRSVTAGRARPPPPGAGDGGKGSSQKRRGPHCARCRSHGVLVPLKGHKKFCNWKNCKCANCQLVMMRRKLMARMIALHRQQDLEAFALQQTGTVPTCSLPGDEVPDPTIKNLGSTNVPLVQSSSSVTGQSATDLLNNFTQGNLPSDSVQASPHSNIQCQWNSNNSMVRSPADASNEIQIQLPSSNELDIPSPLFGAGLASHGGSGRGGSLGPLMAFAPCSSSYSHQQLSFPFGLLTNPKPSTPTSQIYSPNFLVYHHQQQQEQQQVPMQQPYYFQGNRGGETQSAFMPIGQQNPPGHHSGFGHEDSPSPSGVTSPEPFNDIAVLGVASASEPSGIQPAGRAPASHTLRIPLVSRTVQPPYPPPSNAAATTTMSNFHCLPSAASSATQARAFIPLQHALFPQKHTQDSNKN |
| Anj-XP_033117096.1 | MSVGGQIEANQSSKVSISHSGNSGGPPTLLIRSDKPYPRTPKCARCRNHGVVSALKGHKRYCRWRDCVCAKCTLIAERQRVMAAQVALRRQQAQEENEAKELGLYYETTDGAIYAMNGVAVQTHKRSDPYQQLDSPDGKRARLEIIHANSQVSPTTTSAMINGASSPPSSVSSMVDIQAVSPGLHSPTSRDCNNTSPLLSPTISHKNSPKICLADETMSKESRRDREKSRSPPDSPLSLAAKHSGKQPIDTLIQLFPSQKKSVLELILQGCDGDLVQAIEQILSNHHAAQAAHGSSNAFPFPSTSSSNTYVTHRPYLPTTPLGSSGIKSAFSPLSATDRAAMASPTGPFPHMQFAYAPYGRGLALVNPYPPAMFPAFGMRPPTAAEYSFSGLMRDMSGRKDGRQNGTFGAS |
| Anj-XP_033124332.1 | MKTKTSQDVSSSRLPGQPRKHLRTPKCARCRNHGVVSCLKGHKRFCRWRDCQCANCLLVVERQRVMAAQVALRRQQASLPEKDCKLGKTDKTCTVGLTRRTQKTLARSAVLSKDVLQGCRTAKQLRAATNAMQTNRPMFFLPPDVRERMRKRRAFADKELESTMLERECQWSFYYTAHARWTNPTIHRVQQSSSASLALERLTVEPKAHLPPRTAVQRLFPVLIPSAVRTLESGHRKDVTPLEGESIASYSARANEKPVAPFSTVSNSMLKNFHRKPAQLSGKTCHSDVSVHHSVLPVLFQDKQTNENREISTSKETHQQSFEKRFDLSKVNKIKNITNGAAKPPSLLSFSVERIIGLVS |
| Anj-XP_033101076.1 | MMEFSIDQSPIDVQTLNILRGPLTEKGSRKPKCARCRNHGVISWLKGHKRHCRFRDCRCPKCNLIAERQRVMAAQVALKRQQAAEDAIALGLRACSPGGQFVMATGPGVYSENEAKRRKYENDDDDELDYEEEERRLDEKENGVFDEDDHQSSLLLSPVKLTHPCSTSSKSPYTKEKSVSSHSERVPEDVVPPYRPGRLSPLEILMRLFPAQRKTVLELVLQGCNGDLVKAIEHFLAATDTTTTCQEGTQRHSKFSLQSPLSYPGLHGFQPATSDGKYVNEHFKSAFTPLPPSGTPGSNLPFVLAPRGPHHHPLSTDTLLGRTSFFMPGAPGDLTTAAAASRLGLPTFYQTTFSSKLPSTPDGYPRLIFAPYSSCPPECLQCPRPGRLATGSPDASKSPSNLSENGPVVNSTADKE |
| Anj-XP_033105012.1 | MSISGLAQLKSQIGSVGTSNDSQSSTKTEKKIKTRSMPTCSLCRNHGISVLYRGHKRKCRFAMCVCQKCTMTKERRRVMSWHVNEHRANYMAHSRLGEAMAPSTCCANPTSFNHMHPAVYDAWPPTPPGFQALVPYSPECPQPQIGLGLPVPTYYQHYRPTGFLPYDESYPTPSPSPGNNNSAIATFGAEKSSQLLSLSNYYYPTEHTAEGWRPIGVGNGCPTIHVQGQQHNENQLQK |
| Pm-XP_038069302.1 | MNLSGGSIEPQNSDMPASVHHPPTSLQHHHQHHPPTLLLRTEKPYPRTPKCARCRNHGVVSALKGHKRYCRWRDCVCAKCTLIAERQRVMAAQVALRRQQAQEENEAKELGLYYETSDGAIYAMNGVAVQTHKPYDQYHGGTSPESKRPRLEIIRAPSSPSASCASALSPPNPASPNLTVEPHPRTDPDIISQRHVDERVSPPMRLTSPTPSPRGDVPSSPRSEGSGRMLREDYTDRDSYREHNGSGFVDQLSPAQILSFHGRSKGPQRPPVDVLCRLFPAQKRSVLQLVLQGCNGDITQAIEQLLNNQRDEQSTTSMPTSLPVTGAPVTAALPANFPPTATVTTDSHGAYITHRPYLPNSQHLNPGGMKSAFSPLTTAIPVPNVDKAPPTVVASNIPQMRFAYPPYPRGLTLWNPYPPSVIPTAFGVRPATAADYTFSGIMRDLSNGHSGKDCRPNGTFGVGATCD |
| Pm-XP_038069487.1 | MNLSGGSIEPQNSDMPASVHHPPTSLQHHHQHHPPTLLLRTEKPYPRTPKCARCRNHGVVSALKGHKRYCRWRDCVCAKCTLIAERQRVMAAQVALRRQQAQEENEAKELGLYYETSDGAIYAMNGVAVQTHKPYDQYHGGTSPESKRPRLEIIRAPSSPSASCASALSPPNPASPNLTVEPHPRTDPDIISQRHVDERVSPPMRLTSPTPSPRGDVPSSPRSEGSGRMLREDYTDRDSYREHNGSGFVDQLSPAQILSFHGRSKGPQRPPVDVLCRLFPAQKRSVLQLVLQGCNGDITQAIEQLLNNQRDEQSTTSMPTSLPVTGAPVTAALPANFPPTATVTTDSHGAYITHRPYLPNSQHLNPGGMKSAFSPLTTAIPVPNVDKAPPTVVASNIPQMRFAYPPYPRGLTLWNPYPPSVIPTAFGVRPATAADYTFSGIMRDLSNGHSGKDCRPNGTFGVGATCD |
| Pm-XP_038048228.1 | MNSYLVPKLTMLVSKDNSDLDISSPSPGTDSDPGSQTRTKSAPMNPPPRPPTTRKVLRTPKCARCRNHGVVSCLKGHKRYCRWRDCQCTNCQLVVERQRVMAAQVALRRHQATGEDERVAPGKEGAASGAGPPRKVACGAGKGSSGPAGTRKVTLPKGPGHGTTSVSKDILEGCRSKPSRSTAPSSNTPPRPVIFLPPSVSERMRKRRAFADKDLETTMLQRECQWSLMYAAQAGLSRLHPLVDRQHPHQHFRTPAGSDTDLPDQTRQFLRRLFPSHSATALDAALTCSAGNIRLAIEKLVSAYSAASQEGPTPRPLFATGAGLETTPSLPWYLSYPRSKIFKEDEGRMNGAQSDRTETDTPKSSINGIRGEGSVSAFSSLRRRDRGPDSRSRFGSVESENEYSQAFSPGNDRDRNDLADTTPNEIPTNGSLTKRFVKTVSTKRLSFSVDSIMGIN |
| Pm-XP_038069303.1 | MMERPVIDMQTMSLLRGPLTEKGARKPKCARCRNHGVISWLKGHKRHCRFRDCRCPKCNLIAERQRVMAAQVALKRQQAAEDAIAMGLRACSPGAGGSFGMLSTNGPLFGSSDSGDELSPRPMDIADEDGDHHDITEKHMKSESANDKENGRGTPPSGQTERNNNHRHSSSKKGNESLDAFNGFAANFVSNFRPGRLTPIEILTRLFPTQRKAVLELVLQGCSGDLVKAIEHFLSATEDAVQHSPGTASALPPPSISRGVVGQRSALGHEASIGPSGVHGLQTAFRSSLGSEKHPVGSMKSAFTPLPPSSATGSSVPLFFAPRPPHPFHAEALIGRTPLFAPSSVSDLTSSMSSQAIASPRFAFPALHPFHLSGKFPTGGEGYPRLIFAPYATCPPDCIQCPGTRTSMHSEPEKSPSGTTDGSIANSAVDSE |
| Pm-XP_038049237.1 | MTCTKCQISSTYFVHRKVREATNCIKMRKSSKNKRDRSKSSADKQRVEEQGEVVGSTSNSPSTAGHNLTRNVTGSAPNPPPNHGTPTTSRTPRTASPTAGTARAQLPPNRERVTVRTRRYPHCSRCRSHGVLVPIKGHKKTCNWKDCKCHNCQLITARQKLMAKVIALHRQQDMEAVANLQQLPASASHSMAGDEVPDLGVKILSPNNSVPPMPSSLPQGNLMTECQGNPQTNSLPVGPYSSIQYQWNSNNSTHSSPEIGNEIQIQVPQSSETDIPSPIYGTSLASHGSGGRSGSLTPMMAYTPYSNYSHQQLPFPFXLVSTPKLSTATSQIYSPNFLVYHQQQQQQQQQQLQVQMPQYRFQGSSFGSCVGDVQSAFVPVGQQNPATLHTTLAQGDSSPSSSDMTSADAFDEVDVLEVEASGIQTAVKAPASHTLRIPVASRTMQQAFLPASTKDISTMSDFHSLPTAGPSCTQPRAFIPLQNALFPQKHNQDNNKN |
| Pb-KPB_00015970-RA | MATVISEELTVHIIDGDDQDSDVCHTPQSNSDSPIPPPRIKGSPMDTPLSVHPMIPRTQTTRKVLRTPKCARCRNHGVVSCLKGHKRYCRWRDCQCTNCQLVVERQRVMAAQVALRRHQATEGSSPVPGKDGQPAGSGPPRKVPCGAGKGTGGAGARKTAPLRGVGSGSSSVSKDILDGCRSRSSRSSHSTASSPPRPVIFLPPSVSERMRKRRAFADKDLESTMLQRECQWSLMYAAQAGLSRLQPPGTDHHRRASATTSPLIDSHGDIPSQTKHFLRRLFPSLSTTTLDSALRCSGGNLRLAIEKLVTVHSPPSQLVTGTRPDVEHGVVPESTLYLPWYLSYQRNPSLLKENDLRTPSTPFEQRDTNSLVASKTIPKYSMQELRGDGSVSAFTSIHRGGNSLPSSDSRNRFGSVESESEYSRDRDEESIPTNSQDSGGNQTQSKRFLKTTSTSRLSFSVDSIMGKR |
| Pb-KPB_00012322-RA | MIKHNLRELEIEVLHEYLEAKMNGGDRNVGNTQSSSSSRSSRKLNTAAAPSGLTVPAVTAQADASGDASPPCSGGRGRRKKRAPRCSRCRNHGYLVPLKGHKKFCRFKLCKCSYCQLITDRQRVMASQVALKRQQDMEEMVAGCRNASGVVTLPLLKEHPTFKSSCTATSPSLHTVHSGAYINQGNTQMHNFSPNPHPSFQSQWQSNGSTASTPTDINCSENHTFSPLESAETQSSVFGTGLSHSGTFGPMTPFAVYNDSHGSSFPLMINAAKFPPNQMFSSEYLAYQRQLHLQHQMQQQRLRDSSIGNEFGYCNGIQSAFIPLNHQSSSSSGADESLDEDVLEMAREEGGPSGTDDGMGQACQASTNIDRNPGAQRSVLHGFPSPPSTMSDFHSLPVNISVTQAKTFIPLQHALFPQGQTQDKN |
